# *Brucella ceti* Infection in Harbor Porpoise (*Phocoena phocoena*)

**DOI:** 10.3201/eid1612.101008

**Published:** 2010-12

**Authors:** Thierry P. Jauniaux, Cecile Brenez, David Fretin, Jacques Godfroid, Jan Haelters, Thierry Jacques, Francis Kerckhof, Jan Mast, Michael Sarlet, Freddy L. Coignoul

**Affiliations:** Author affiliations: Veterinary College, Liege, Belgium (T.P. Jauniaux, C. Brenez, M. Sarlet, F.L. Coignoul);; Royal Belgian Institute of Natural Sciences, Brussels, Belgium (T. Jauniaux, J. Haelters, T. Jacques, F. Kerckhof);; Veterinary and Agrochemical Research Centre, Brussels (D. Fretin, J. Mast);; Norwegian School of Veterinary Science, Tromsø, Norway (J. Godfroid)

**Keywords:** Bacteria, zoonoses, Brucella ceti, cetaceans, harbor porpoise, brucellosis, dispatch

## Abstract

We describe *Brucella* sp. infection and associated lesions in a harbor porpoise (*Phocoena phocoena*) found on the coast of Belgium. The infection was diagnosed by immunohistochemistry, transmission electron microscopy, and bacteriology, and the organism was identified as *B. ceti*. The infection’s location in the porpoise raises questions of abortion and zoonotic risks.

In cetaceans, *Brucella* spp. infections and related lesions have been found in bottlenose dolphins (*Tursiops truncatus*) (*1*), striped dolphins (*Stenella coeruleoalba*) (*2**–**5*), Atlantic white-sided dolphins (*Lagenorhynchus acutus*) (*6**,**7*), common dolphins (*Delphinus delphi*) (*6**,**8*), harbor porpoises (*Phocoena phocoena*) (*6**,**9*), and a minke whale (*Balaenoptera acutorostrata*) (*6*). Recently, *B. ceti* was described as being the cetacean *Brucella* sp. strain that infects dolphins (*10*). We report a case of *B. ceti* infection and associated lesions in a harbor porpoise found on the coast of Belgium in 2008.

## The Study

**Figure 1:**
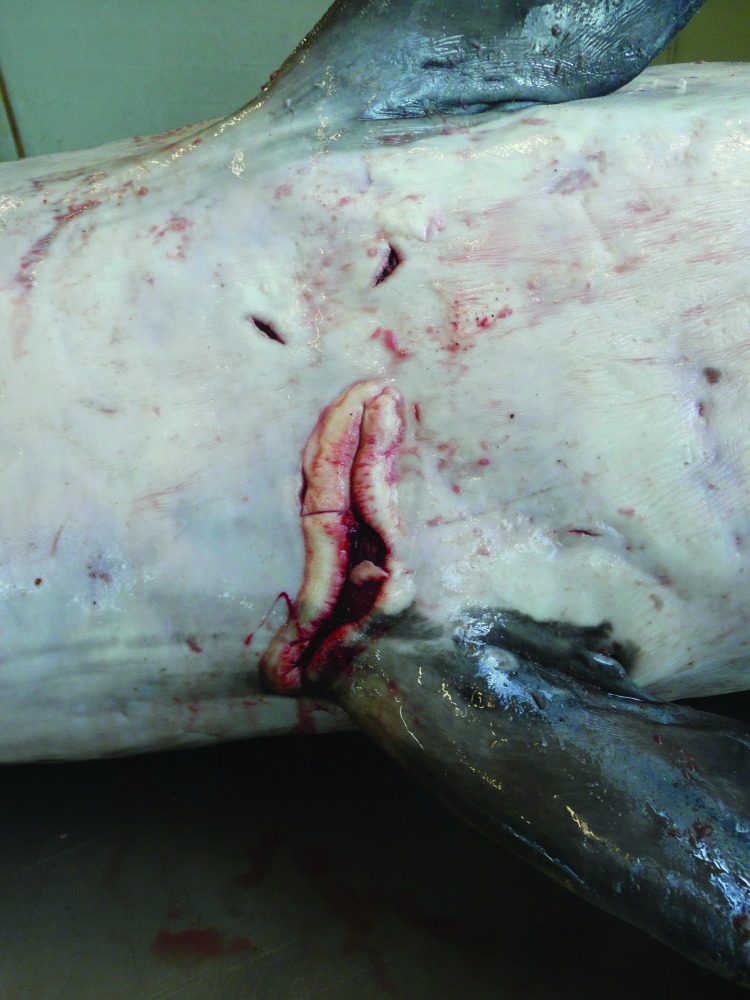
Longitudinal ulcer between flippers of a harbor porpoise (*Phocoena phocoena*) with *Brucella ceti* infection, Belgium, 2008.

An adult female harbor porpoise died on the coast of Belgium in 2008, and a necropsy was immediately performed by the Marine Animals Research and Intervention Network (Belgium). The most relevant findings (Table) were emaciation and multiple large skin ulcers (acute to chronic) around the genital split and between flippers (Figure 1). Internally, mild to severe nematode infestation (of the right ventricle, pulmonary blood vessels, airways) was associated with acute pulmonary thrombi and severe acute necrotizing pneumonia. The liver was enlarged and yellowish with multiple 1–2-mm red to dark red spots. The uterus was dilated with a larger left uterine horn and prominent congested blood vessels; a corpus luteum cyst was present in the left ovary. Microscopic examination showed severe, acute, necrotizing pneumonia and interstitial subacute to chronic pneumonitis with arteritis (mostly associated with lungworms); multiple foci of acute coagulative necrosis in the liver; and mild, multifocal, nonsuppurative meningitis. The mammary gland contained numerous small acini with small amounts of milk in the acini and ducts. Infiltrate of mononuclear cells under the endometrium suggested endometritis.

**Table:** Postmortem findings in a harbor porpoise infected with *Brucella ceti*, Belgium, 2008*

Sample	Necropsy	Histologic	Immunohistochemistry†	Electron microscopy	Bacteriologic
Brain	NS	Slight subacute meningitis	Glial cells	NT	*Brucella* isolate
Uterus	Congestion and hyperplasia	Slight subacute endometritis	Mononuclear cells under the endormetrial epithelium	NT	NT
Mammary gland	Congestion	Well-developed acini with milk	Mononuclear cells between acini, acinar cells, and milk	NT	NT
Liver	Multifocal red to dark red spots	Coagulative necrosis	Mononuclear cells in portal areas	NT	NT
Lungs	Multifocal acute necrotizing thrombo-embolic pneumonia	Acute purulent pneumonia, severe subacute to chronic interstitial pneumonitis	Mononuclear cells and nematode larvae	NT	*Brucella* isolate
Lymph nodes	Hyperplasia	Lymphoid depletion	Mononuclear cells near the capsule	NT	NT
Skin and genital split	Multiple acute to chronic ulcers	Acute ulcerative dermatitis with ballooning degeneration	Balloon degenerated epithelial cells and inflammatory infiltrate	Intracellular coccoid bacteria (genital ulcer)	NT
Spleen	Hypoplasia	Lymphoid depletion	Mononuclear cells near the splenic capsule and between splenic corpuscles	NT	NT

*NS, not significant; NT, not tested. †With polyclonal antibody against *B. melitensis.*

**Figure 2:**
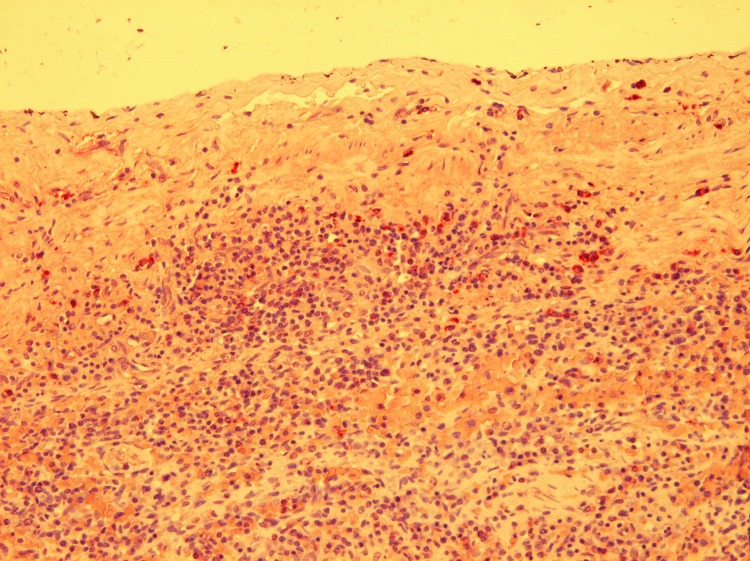
Positive immunohistochemical staining in mononuclear cells below the splenic capsule in a harbor porpoise (*Phocoena phocoena*) with *Brucella ceti* infection, Belgium, 2008. Original magnification ×200.

Immunohistochemical investigation, using polyclonal antiserum obtained from a rabbit experimentally infected with *B. melitensis*, showed diffuse intracytoplasmic positive staining for *Brucella* spp., primarily in mononuclear and inflammatory cells on various tissues (spleen [Figure 2], lymph nodes, lung, uterus, liver, pancreas, and brain), in lesions, in lungworms, and in mammary gland acini and milk. By transmission electron microscopy, large numbers of relatively small (diameter 380–450 nm) intracellular coccoid bacteria that suggested *Brucella* spp. were observed in the genital ulcer. A *Brucella* sp. isolate was obtained from brain and lung tissue. The strain grew on *Brucella* agar supplemented with 5% horse serum in the presence of basic fuchsine, thionine, and growth on safranin O. CO_2_ was not required for growth, and H_2_S was not produced. The isolates showed catalase, oxidase, and urease activity. This biotype profile is in agreement with the strain type profile of *B. ceti* (*10*). Multilocus variable number tandem repeat analysis (MLVA) typing, which used MLVA panel 1 (8 minisatellite loci: bruce06, bruce08, bruce11, bruce12, bruce42, bruce43, bruce45, and bruce55, which are useful for species identification), showed that the strains belong to genotype 23 (*11*). MLVA panel 2 was split into 2 groups, panels 2A and 2B, comprising 3 (bruce18, bruce19, bruce21) and 5 (bruce04, bruce07, bruce09, bruce16, bruce30) markers, respectively (*12*). Using panel 2A, we obtained the same profile as the one described for all *B. ceti* strains isolated from porpoises (*11*), whereas panel 2B showed a new genotype (bruce04: 6 repeats, bruce07: 6 repeats, bruce09: 3 repeats, bruce16: 7 repeats, bruce30: 6 repeats), closely related to genotypes ascribed to *B. ceti* strains isolated from porpoises mainly stranded in Scotland (*11*). The new genotype identified by panel 2B is possibly associated with southern North Sea porpoises. However, panel 2B contains the more variable loci, and this panel has been given a lower weight in clustering analysis (*12*).

The results suggest a bacteremia associated with *B. ceti*. The infection was suspected after examination by electron microscopy and confirmed by bacteriologic and immunohistochemical investigations; finally, the bacterium was identified as *B. ceti*. In Europe, most reported cases of cetacean brucellosis have been reported from the coasts of Scotland and England and found in striped dolphins, Atlantic white-sided dolphins, common dolphins, harbor porpoises, and a minke whale (*2**,**3**,**6**,**7**,**9*). Meningoencephalitis associated with *Brucella* spp. infection has been reported for striped dolphins (*2**,**3*) and 1 Atlantic white-sided dolphin (*7*). Necrosis of spleen, liver, and lymph nodes associated with *Brucella* spp. infection has also been reported for Atlantic white-sided dolphins (*6*). In porpoises, *Brucella* spp. have been isolated from different organs without associated pathologic changes other than coagulative necrosis of the spleen (*6*) and a testicular abscess (*9*). Finally, in the minke whale, foci of liver necrosis and inflammation were consistent with lesions caused by *Brucella* spp (*6*). In our study, the enlarged uterine horn, the corpus luteum cyst, and the presence of milk in mammary acini suggested recent pregnancy, and the positive immunolabeling of the endometrium raised the question of a possible abortion. Indeed, *Brucella* spp. are known to be responsible for abortions in terrestrial mammals, Brucella spp.–induced abortions have been described in 2 bottlenose dolphins with associated placentitis (*1*), and *Brucella* spp. have been isolated from an aborted bottlenose dolphin fetus (*13*). *Brucella* antigens were detected in the placenta of a stranded striped dolphin with a 7-month-old dead fetus (*5*). In addition, vaginal lithiasis suspected to be the result of ossification of aborted fetuses in 2 common dolphins positive for *Brucella* spp. in the uterus has been reported (*8*).

## Conclusions

In the present case, a final conclusion cannot be drawn with respect to a possible abortion. Identification of *B. ceti* in milk (as in the present study) and in fetal tissues and secretions of a pregnant dolphin suggest that *B. ceti* has tropism for placental and fetal tissues and that it can be shed externally (*4*). This finding suggests potential vertical and horizontal transmission to newborns (*4*). Nevertheless, indirect transmission through parasites should not be excluded because *Brucella* spp. have been identified from lungworms (*14*). In addition, the observation of *Brucella* spp. antigens in milk and in skin ulcers may represent routes of bacterial transmission between individual animals and raises the question of the risk for transmission to a person handling the cetacean (e.g., on the beach or in a rehabilitation center). All persons handling wild or captive marine mammals (alive or dead) or samples collected from the mammals should be aware of such risks and take necessary precautions. To date, 4 cases of human infection with *Brucella* spp. from marine mammals are known. One was mild and uncomplicated in a laboratory worker; however, the 3 other cases were severe naturally acquired without direct contact with marine mammals but with a history of eating raw fish or shellfish (*15*).

We emphasize that further investigations are needed to improve knowledge of the prevalence, the impact on individual cetaceans and populations, and the zoonotic potential of marine mammal brucellosis. The zoonotic risk should be taken into account by all persons in contact (direct or indirect) with marine mammals. Finally, the present case confirms the need for careful monitoring and complete postmortem examinations of stranded marine mammals.
